# Advocacy for outpatient cardiac rehabilitation globally

**DOI:** 10.1186/s12913-016-1658-1

**Published:** 2016-09-06

**Authors:** Abraham Samuel Babu, Francisco Lopez-Jimenez, Randal J. Thomas, Wanrudee Isaranuwatchai, Artur Haddad Herdy, Jeffrey S. Hoch, Sherry L. Grace, in conjunction with the International Council of Cardiovascular Prevention and Rehabilitation (ICCPR)

**Affiliations:** 1 Department of Physiotherapy, School of Allied Health Sciences, Manipal University, Manipal, 576104 Karnataka India; 2 Preventive Cardiology Program, Division of Cardiovascular Diseases, Mayo Clinic, Rochester, MN USA; 3 Centre for Excellence in Economic Analysis Research, Li Ka Shing Knowledge Institute, St. Michael’s Hospital, 30 Bond Street, Toronto, M5B 1 W8 ON Canada; 4 Institute of Health Policy, Management and Evaluation, University of Toronto, 155 College Street, Toronto, M5T 3 M7 ON Canada; 5 Institute of Cardiology of Santa Catarina, Universidade e do Sul de Santa Catarina, Palhoça, Brazil; 6 School of Kinesiology and Health Science, York University, Bethune 368, York University, 4700 Keele Street, Toronto, M3J 1P3 ON Canada; 7 Toronto Western Hospital, GoodLife Fitness Cardiovascular Rehabilitation Unit, University Health Network, Toronto, ON Canada

**Keywords:** Cardiovascular disease, Reimbursement, Cardiac rehabilitation, Insurance

## Abstract

**Background:**

Cardiovascular diseases (CVD) are the leading cause of death globally. Cardiac rehabilitation (CR) is an evidence-based intervention recommended for patients with CVD, to prevent recurrent events and to improve quality of life. However, despite the proven benefits, only a small percentage of those would benefit from CR actually receive it worldwide.

This paper by the International Council of Cardiovascular Prevention and Rehabilitation forwards the groundwork for successful CR advocacy to achieve broader reimbursement, and hence implementation.

**Methods:**

First, the results of the International Council’s survey on national CR reimbursement policies by government and insurance companies are summarized. Second, a multi-faceted approach to CR advocacy is forwarded. Finally, as per the advocacy recommendations, the economic impact of CVD and the corresponding benefits of CR and its cost-effectiveness are summarized. This provides the case for CR reimbursement advocacy.

**Results:**

Thirty-one responses were received, from 25 different countries: 18 (58.1 %) were from high-income countries, 10 (32.4 %) from upper middle-income, and 3 (9.9 %) from lower middle-income countries. When asked who reimburses at least some portion of CR services in their country, 19 (61.3 %) reported the government, 17 (54.8 %) reported patients pay out-of-pocket, 16 (51.6 %) reported insurance companies, 12 (38.7 %) reported that it is shared between the patient and another source, and 7 (22.6 %) reported another source.

**Conclusions:**

Many patients pay out-of-pocket for CR. CR reimbursement around the world is inconsistent and insufficient. Advocacy campaigns forwarding the CR cause, supported by the relevant literature, enlisting sources of support in a unified manner with an organized plan, are needed, and must be pursued persistently.

**Electronic supplementary material:**

The online version of this article (doi:10.1186/s12913-016-1658-1) contains supplementary material, which is available to authorized users.

## Background

Cardiovascular disease (CVD) is the leading cause of death globally, [1] and the burden of CVD is growing [2]. Consequently, CVD accounts for 10 % of disability-adjusted life years (DALYs) lost worldwide; 10 % of DALYs lost in low and middle-income countries, and 18 % of DALYs lost in high-income countries [3, 4]. According to the Global Burden of Disease Study, CVD was the leading cause of disability-adjusted life years lost in 2010 [4]. In the same year, years lived with disability due to ischemic heart disease was 8795 for all ages, or 128 per 100,000 [5].

Cardiac rehabilitation (CR) is an outpatient chronic disease management program for the secondary prevention of CVD. The World Health Organization has defined CR as the “sum of activities required to influence favorably the underlying cause of the disease, as well as the best possible physical, mental and social conditions, so that they may by their own efforts, preserve or resume when lost, as normal a place as possible in the society” [6]. The core components of CR include baseline patient assessment, nutritional counseling, risk factor modification, psychosocial interventions, physical activity counseling and exercise training [7–13].

The substantive clinical benefits of CR have recently been summarized [14, 15]. In brief, in high-income countries, CR is shown to reduce morbidity and mortality by 25 % [15]. In low and middle-income countries, CR participation is associated with significant improvements in lipids, body mass index, blood pressure, as well as quality of life, and functional capacity [14, 16].

**Fig. 1:**
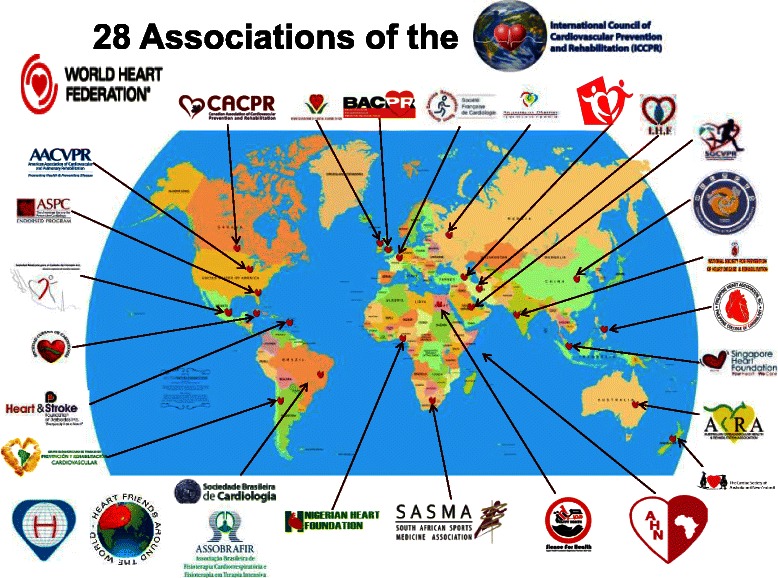
Member association of the International Council of Cardiovascular Prevention and Rehabilitation

Unfortunately, CR is grossly under-developed around the globe [17]. While the reasons are multi-factorial, arguably the chief reason is lack of service reimbursement. Accordingly, the International Council of Cardiovascular Prevention and Rehabilitation (www.globalcardiacrehab.com; Fig. 1) set out to develop a resource for CR professionals and associations to advocate for CR reimbursement. This conceptual paper first describes the scope of the problem of low use of CR and reimbursement. Results from a survey undertaken by the International Council regarding CR reimbursement by government and insurance companies in various regions of the globe are presented. Second, a 6-step approach to advocating for CR is provided. The approach starts with demonstration of the scale of the CVD problem, and then supporting evidence. Accordingly, the paper concludes with this needed information, summarizing the economic impact of CVD and the economic benefits of CR, to support CR advocacy efforts.

### Significant Gap in cardiac rehabilitation availability and participation

CR services are scarce. Specifically, CR is available in only 38.8 % countries worldwide: 68.0 % of high-income, 28.2 % of middle-income and 8.3 % of low-income countries [17]. Where CR programs are available, it has been seen that most, if not all countries have insufficient capacity to treat indicated patients [17]. The number of CR programs per inhabitant (which we refer to as CR density), is a crude estimate of the number of patients who might have access to CR in each country [18]. Based on national and regional surveys in high-income countries, CR density ranges from one program per 100,000 to one program per 300,000 inhabitants [19–21]. In middle-income countries, CR density ranges from 0.9 to 6.4 million inhabitants per program [19]. No low-income country is known to have more than 1 CR program [17].

Accordingly, participation in CR is also low [22–24]. For example, the European Action on Secondary Prevention by Intervention to Reduce Events III Survey showed that only 36.5 % of 8845 indicated patients from 22 European countries (19 of which were high-income) attended CR [25]. Findings from the European CR Inventory Survey revealed that CR enrolment rates >50 % were seen in only 3 (10.7 %) countries while rates <30 % were reported in 15 (53.6 %) of the 28 countries [26]. In the United States, the largest study ever on CR utilization among 601,099 Medicare beneficiaries eligible for CR demonstrated that only 12.2 % participated [27]. The international Stabilisation of Atherosclerotic Plaque by Initiation of Darapladib Therapy trial revealed that majority of the participants (65 %) had never participated in CR [28]. The International Council on Cardiovascular Prevention and Rehabilitation argues that lack of CR provision, and hence patient access, is a direct function of lack of reimbursement.

### Reimbursement of cardiac rehabilitation

Coverage of services is tantamount to increasing delivery. Unfortunately, very little is known about reimbursement for CR services around the globe. In order to better understand this issue, several authors (AB, FL, SG) designed an online survey assessing CR reimbursement on behalf of the International Council of Cardiovascular Prevention and Rehabilitation. Items assessed the nature of coverage for CR services by government and healthcare insurance companies, using a similar approach as described previously [18, 29]. The survey was reviewed by experts in the area of CR. Then, it was emailed to the member organizations of the International Council of Cardiovascular Prevention and Rehabilitation (Fig. 1), who were asked to complete it and forward it to their CR colleagues from different countries (i.e., snowball sampling). Respondents were asked to contact the top 3 health insurers in their country on the basis of premiums collected and to consult official government statistics or academic publications to provide accurate reimbursement data.

This was a cross-sectional study. Thirty-one responses were received, representing 25 countries (or 34 % of all 83 countries that offer CR globally) [17]. Fifty-eight percent of respondents were from high-income, 32 % upper middle-income, and 10 % were from lower middle-income countries. It must be conceded that the generalizability of responses is unknown, the number of responses was low, and the source of estimates is unknown (e.g., whether government statistics were accessed and the number of insurance providers successfully contacted). Therefore the results presented herein, while providing initial valuable information on CR reimbursement internationally, should be interpreted with caution. Moreover, results should not be considered representative of low-income countries.

When asked who reimburses CR in their country (respondents were asked to check all that apply), 61 % reported the government, 55 % reported patients pay out-of-pocket, 52 % reported insurance companies, 39 % reported that it is shared between the patient and another source, and 23 % reported another source (e.g., public hospitals only, subsidized by Heart Foundation, insurance coverage by some companies only).

#### Government reimbursement

Government-reimbursed indications for CR were most often myocardial infarction, coronary artery bypass graft surgery and percutaneous coronary intervention (all 100 %), followed by heart failure, valve surgery/procedures, and heart transplant (all 87 %), as well as stable angina (73 %), and rhythm devices (60 %). Respondents reported that a mean of 32.0 (standard deviation; SD = 12.9) CR sessions were covered by government for each patient, and that a mean of 91.5 % (SD = 19.2 %) of the total CR program cost was covered by government (i.e., no deductible or out-of-pocket fee). Almost one-fifth of respondents reported that the government limits the components of CR covered.

Aspects of CR which were reimbursed most often included supervised exercise (93 %), followed by dietary counselling, mental health/psychological support, smoking cessation, hypertension control and hyperlipidemia control (all 80 %), education (73 %), weight control (67 %), and return-to-work/occupational therapy (53 %). When asked whether the government specified the type of professional treating the cardiac patient to be eligible for CR funding, over half responded yes; the type of professional was most often a cardiologist, nurse or physiotherapist (each 99 %).

#### Insurance company reimbursement

Reimbursed indications for CR were synonymous with those reported with government funding. Respondents reported that mean of 22.2 (SD = 15.4) sessions were covered, and that mean of 48.3 % (SD = 50.5 %) of the total CR program cost was covered by private healthcare insurance. Where the patient paid some money toward CR, the average cost was USD$17.5 (SD = 6.9)/session or USD$345.0 (SD = 38.2)/program. Some respondents commented that the insurance companies only covered physical activity and exercise training.

### Advocacy for cardiac rehabilitation

Advocacy is “the act or process of supporting a cause or proposal” [30]. It involves the art of communication by an individual or group, often on behalf of others, with the purpose of supporting an idea or cause. Effective advocates influence public policy, laws and budgets by using facts, personal stories, their relationships, the media, and messaging to educate government officials, policy-makers and the general public about the importance and the potential impact of the idea or cause they are supporting. When applied in the healthcare setting, advocacy is carried out at various levels by a variety of people—patients, providers, healthcare advocacy groups, healthcare industry representatives, and others. Healthcare advocacy work is important because the voice of advocates can help shape and implement important and beneficial healthcare policies and practices.

Policy-makers face a challenging task of decision-making while being inundated with a large amount of data, opinions, and requests. They must maintain a delicate balance between what is best for individuals and what is best for society. Effective advocates assist policy-makers in these balancing efforts by helping to clarify and simplify the complexities of issues that surround a given idea or cause. Advocates generally speak with a unique degree of authenticity, as they bring personal stories and experiences with them that link them to the cause for which they advocate.

The cause for reimbursement and delivery of CR can be bolstered by common advocacy pathways. These including the upcoming Sustainable Development Goals, in particular the third proposed goal regarding health, specifically reducing premature mortality from non-communicable diseases through prevention and treatment by one-third by 2030, as well as achieving universal health coverage to promote access to essential medicines [31, 32]. These current windows of opportunity represent areas where CR advocacy could find synergy and hence greater success.

#### Cardiac rehabilitation-specific advocacy

Despite the large amount of evidence [15, 33, 34] showing the benefits of CR services to eligible patients, advocacy has been challenging. CR services are beneficial, yet relatively simple and low-cost, when compared to other services in the field of cardiovascular medicine. Arguably then, CR services have not generally attracted much attention from administrators, clinicians, and even patients, given the “low-tech”, lower budget nature of CR services.

In the past three decades, CR professionals from various countries around the world have been involved as advocates on behalf of their patients, helping to shape and implement important healthcare policies for the provision of CR to eligible patients. Advocacy messaging has been primarily based on what is in the best interests of eligible patients—to promote the delivery of CR services to the large number of individuals who are eligible for such services each year.

Components of a successful CR advocacy program are multi-faceted, and include the following:

- **A just “cause”:** The strength of an advocacy program is dependent largely on the evidence that CR services provide strong, positive benefits for individuals and/or society.
- **Publications to support the cause:** CR advocacy is strengthened by research studies and other scholarly works that have been published in respected, peer-reviewed scientific publications.
- **Key sources of support:** Advocacy is dependent on key voices of support from the general population, key national healthcare leaders, key healthcare organizations, key healthcare policy-makers (i.e., someone in an influential position to bring about a solution or with a personal connection to the issue), and high visibility public figures who are “champions” for the cause, giving a face and a sense of personal impact to the cause.
- **Unified support:** Successful CR advocacy depends on unified support—either for clinical practice or policy reasons—from leading professional organizations in the field of cardiology.
- **Organized plan:** An organized plan is critically important to CR advocacy efforts; a plan that includes goals, strategies and tactics to address scientific, policy, financial, and communication needs, among other things.
- **Persistence:** CR advocacy efforts take time and patience, given the resistance to change that exists at all levels of the decision-making process, from patients and providers, to policy-makers and healthcare leaders.

Communications methods are critically important in the work of advocacy towards CR. The key to coverage negotiations is identifying the needs and benefits to the patient, government and/or insurance companies. By providing an explanation of the services and benefits of CR for insured clients, coverage may be considered by governments and insurance companies. Key points for communication in a CR advocacy campaign are presented in Table 1.

**Table 1:** Communication methods for a cardiac rehabilitation advocacy campaign

Method of communication	Description
Key messages	This includes important messages that should be repeated often, and be easy to understand in order to gain support
Letter-writing and/or phone call campaigns	This includes campaigns towards policy makers which are well-controlled and coordinated.
Meetings with policy-makers, either in private or in public	This provides prime opportunity to personalize the cause they are supporting by sharing their perspective, story, and passion.
Media messaging on the issue (including social media and websites)	This includes sharing stories on CR topics (e.g., patient’s personal story of triumphs or struggles with heart disease, new findings of scientific significance to the field, or expert opinions about urgent public health concerns or heart-related illnesses of public figures) through various media sources

*Abbreviation*: *CR* cardiac rehabilitation

In countries where advocacy work has not yet resulted in insurance coverage policies for CR, healthcare systems could consider alternative CR models at least for the short-term, including home-based CR, mobile technology tools, and other forms of lower-cost delivery models (e.g., outdoor Asociacion Cardiovascular Centrooccidental CR program in Barquisimeto, Venezuela).

CR professionals have begun advocating for broader CR delivery and reimbursement. Relenting efforts have paid off in several countries such as Iran, Qatar, the United States and United Kingdom. A summary of these success stories is given in Table 2, and include CR-supportive funding policy and program initiation in countries where CR did not exist. For a detailed description, please refer to the online Additional file 1.

**Table 2:** Summary of success stories from four countries across the globe

Country	What did they do?	What did they achieve?
Iran	Developed a CR research center Enhanced research Discussion and seminars with policy-makers & insurance companies	Directive from the Ministry of Health that all components of CR will be reimbursed by insurance companies Improved CR attendance
Qatar	Developed clinical services and formed a CR planning committee Collaborating with other organizations for phase 3 CR	Formed the working group of Qatar Association for Cardiovascular Prevention and Rehabilitation Improved CR referrals
United Kingdom	Evidence-based campaigning for reimbursement Emphasis from national guidelines on CR and formation of standards for delivery of CR	Created a National Commissioning Guide and Tool-kit to fund CR Improved CR referrals
United States of America	AACVPR developed performance measures for CR Provided evidence-based campaigns from long-term studies Conducted government sponsored projects showing cost- effectiveness of CR	Developed performance measures of CR HF included under indications for CR referral State health plans to cover essential health benefits related to CR

*Abbreviations*: *CR* cardiac rehabilitation, *AACVPR* American Association of Cardiovascular and Pulmonary Rehabilitation, *HF* heart failure

To promulgate successful advocacy efforts, organizations like the American Association of Cardiovascular and Pulmonary Rehabilitation [35] and World Heart Federation [36], have provided advocacy tools that can be found online. An ICCPR toolkit and accompanying pamphlet can be found in Additional files 2 and 3, respectively. Below evidence supporting the need for CR and its benefits, as per the first 2 CR-specific advocacy components (namely the “cause” and the sources of information to support it) are provided below.

### The need for cardiac rehabilitation

#### Economic impact of cardiovascular disease

In addition to its’ impact of on mortality and morbidity as mentioned in the introduction, CVD is also associated with a significant economic burden. Focusing on only direct cost (i.e., costs associated with hospital inpatient care, outpatient physician visits, and medications), CVD has the highest total costs of any health condition [37].

In the European Union, CVD was estimated to cost over €106,156,940,0002009 for health care-specific costs which included five categories: primary care, outpatient care, emergency, inpatient care, and medications. Non-health care costs were estimated at €26,963,326,000 due to mortality, €18,873,665,000 due to morbidity, and €43,560,202,000 in informal care. Overall, the cost of CVD to EU was approximately €196 billion annually in 2009 [38] which is a steep increase from €169 in 2003 [39].

A similar picture emerges in North America. In the United States, CVD also accounts for a large share of total spending. The average annual cost per person attributable to CVD is $4734 (2005 American dollars) [40]. By 2030, 40.5 % of the American population is projected to have some form of CVD, and total direct costs are projected to reach more than $800 billion in 2008 American dollars (from $273 billion in 2010). The indirect costs due to productivity loss are estimated to increase to $276 billion in 2030.

In low and middle-income countries, the burden of CVD is of epidemic proportions [41, 42]. Using economic growth models, which assess the effect of chronic diseases on national income, the estimated losses because of coronary heart disease, stroke, and diabetes ranged from $20 to 30 million in Ethiopia and Vietnam to almost $1 billion in larger countries such as India and China [41]. In the scenario with no support to reduce risk of chronic diseases, an estimated $84 billion of national income (American dollars) will be lost to heart disease, stroke, and diabetes alone in 23 selected low and middle-income countries between 2006 and 2015, namely China, India, Russia, Brazil, Indonesia, Mexico, Turkey, Pakistan, Thailand, Bangladesh, Ukraine, Egypt, Argentina, Burma, Iran, Poland, South Africa, Philippines, Colombia, Vietnam, Nigeria, Ethiopia, and Democratic Republic of the Congo [41].

From an individual’s perspective, CVD poses a great economic burden on the family and the community. This is not only due to productivity loss, but also care costs. Studies indicate that CVD drives approximately 10 % of affected families into poverty in a low and middle-income country like India [16]. Moreover, due to disability caused by CVD, individuals may require assistance with activities of daily living (which costs money), as well as financial support if their productivity decreases.

#### Economic impact of cardiac rehabilitation

Overall, CR has consistently shown to be either cost-saving or to be cost-effective regardless of the country where it was examined, the perspective used, the costs included, and the year when it was analyzed [33]. In fact, European studies have demonstrated that CR may actually be a cost-saving intervention [25]. A cost-effectiveness analysis using pooled data from randomized clinical trials and cohorts demonstrated that CR would cost $4950 per year of life saved [26]. Systematic reviews of all the available evidence show savings of $12,000/CR patient over 5 years, to $9200 per quality-adjusted life year [33]. To put this in perspective, Table 3 shows cost-effectiveness values for common treatments and procedures for the secondary prevention of CVD.

**Table 3:** Cost-effectiveness estimations for different interventions in patients with coronary artery disease

Author (year)	Intervention	Patient population	Estimated savings
Ades et al. (1997) [46]	CR versus with other post-MI treatment interventions	Post MI or revascularization	CR was found to result in savings of 2,130 $/YLS in 1980, which was projected to be 4,950 $/YLS for 1995
Johanneson et al. (1997) [47]	Statins (i.e., Simvastatin) versus no statins	Angina or MI	Simvastatin use resulted in $3,800 to $27,400 cost per year of life gained
Cleland et al. (1997) [48]	CABG + Medical therapy + aspirin versus CABG + medical therapy + aspirin + statin versus medical+aspirin+statin versus medical + aspirin	Chronic stable angina	$36,709, $55,156 and $23,730 per QALY for each comparison over 5 years
Chan et al. (2007) [49]	High intensity versus low intensity statin	Acute coronary syndrome, Chronic coronary disease	From $20,000 to $35,000 if cost difference of statins is between $2 and $3.50 From $70,000 to $125,000 if cost difference of statins is between $2 and $3.50
Dendale et al. (2008) [50]	CR versus no CR	Post PCI	Reduction in total health care costs with CR (€4,862/patient versus €5,498 Euro/patient)
Weinbtraub et al. (2008) [51]^a^	PCI and medical therapy versus Medical therapy alone	Stable angina	$168,000 to $300,000 per QALY gained with PCI
Wilson et al. (2012) [52]	Smoking cessation with varenicline plus counseling versus counseling only	CVD	Savings ranging from €5151 - €6120 per QALY gained
Smith et al. (2013) [53]	Implantable cardiac defibrillator versus no defibrillator	Primary prevention of sudden death in patients with left ventricular ejection fraction <40% (ischemic and non-ischemic)	€43,993 per QALY gained compared to no defibrillator

Abbreviations: *$/YLS* dollars per year of life saved, *CVD* cardiovascular disease, *MI* myocardial infarction, *PCI* percutaneous coronary intervention, *QALY*, quality-adjusted life year

^a^COURAGE (Clinical Outcomes Utilizing Revascularization and Aggressive druG Evaluations) trial

Because CR has been demonstrated to decrease total mortality, cardiovascular mortality, cardiovascular events, procedures, and re-hospitalizations, and has shown to improve quality of life, the denominator in the cost-effectiveness equation is generally as good, or better, than many other cardiovascular interventions [33, 34, 43, 44]. In addition to these benefits, CR has also been reported to increase return to work [45]. Those values are presumably stable across countries and geographic regions, as the benefit of CR is expected to be similar (as long as the program is of good quality and similar to the programs used in the primary studies). On the contrary, the cost component of a cost-effectiveness analysis will vary from country to country, because the cost of major components of CR can be significantly different across geographic regions. For example, major components like the salary for nurses, physicians, and other healthcare providers are significantly higher in high-income countries when compared to low and middle-income countries. Likewise, costs related to use of physical space and other costs related to the overall expenses of CR can also be significantly higher in a high-income country.

Therefore, as the measure of effectiveness is expected to be similar but costs are expected to be lower in low and middle-income countries, it is safe to assume that the estimates of cost-effectiveness for CR will probably be more favorable in low and middle-income countries. For example, if the overall cost of CR in a low and middle-income country is only one-half of the total cost in a high-income country, the already favorable estimates for cost-effectiveness may actually become cost-saving. This suggests that in a low and middle-income country, not providing CR might actually be more expensive to payers and to society than providing CR. This can occur because the lack of a medical intervention (i.e., CR) meant to prevent adverse events and procedures would lead to major expenses, making the lack of CR more expensive than offering it itself. We do concede however that more data in this area is needed.

## Conclusions

The need for CR has been established, and the economic benefits of CR provision have been demonstrated. Given the evidence of CR benefits for patient health and vocational outcomes, greater provision of these services through coverage may result in fewer recurrent cardiac events and associated hospitalizations, fewer revascularization procedures, as well as greater return-to-work and productivity. Given that CR is cost-effective, greater provision of such programs may be of significant economic benefit to government, insurance companies, the private sector and individuals.

It is imperative to advocate for reimbursement of CR services so that availability and affordability for patients will be greatly increased. An ancillary consequence will be that healthcare providers will be motivated to train and work in the field of CR, which will also enable greater implementation. It is incumbent upon CR associations to advocate for CR coverage, using the strategies forwarded herein.

## Additional file

Additional file 1: Success stories. (DOCX 23 kb)
Additional file 2: Cardiac rehabilitation pamphlet. (PDF 1.09 mb)
Additional file 3: Cardiac rehabilitation advocacy tool kit. (PDF 229 kb)

## Abbreviations

CR: Cardiac rehabilitation
CVD: Cardiovascular disease
DALYs: Disability-adjusted life years
ICCPR: International Council for Cardiovascular Prevention and Rehabilitation
SD: Standard deviation
WHO: World Health Organization
