# Supplementary material for: Advocacy for outpatient cardiac rehabilitation globally
Source: BMC Health Serv Res. 2016 Sep 6;16:471. doi: 10.1186/s12913-016-1658-1 (PMC5013580; doi:10.1186/s12913-016-1658-1)
Supplement: Additional file 1: — Success stories. (DOCX 23 kb) [file 12913_2016_1658_MOESM1_ESM.docx]

**SUPPLEMENTARY MATERIAL**

**Four cardiac rehabilitation advocacy success stories from around the world**

There have been individuals and groups working in various parts of the world, LMIC and high-income, to promote and increase the provision of CR in their respective countries. The following section highlights these successes initiating CR from Iran and Qatar. In addition, we also highlight significant contributions from the United Kingdom (UK) and US in achieving broader reimbursement of CR.

Iran

The first CR program in Iran was established in 1996 at the Isfahan Cardiovascular Research Centre; after that, several CR programs were established in Iran. With a research mandate, this unit was later developed into the CR Research Centre (CRRC) in 2010 as part of the Cardiovascular Research Institute. The CRRC’s long-term goals are: (1) Improving existing knowledge about CR and secondary prevention of CVD; (2) Conducting clinical, epidemiologic and basic research in the secondary prevention and CR fields; (3) Delivering PhD training through research courses; and (4) Holding training courses for national and international groups to teach them how to establish CR programs as well as how to deliver different CR models.

One of the main patient-related barriers to participation in CR is financial in most countries in the world; Iranian patients faced the same problem. This led to high rates of non-adherence or drop-out, or patients failing to even enroll in CR. Therefore, several sessions with the insurance companies and the Ministry of Health (MOH) were held to advocate for CR. Its importance from an economic, developmental, and social perspectives where highlighted, in addition to the health benefits.

We organized meetings as seminars/workshops or face-to-face discussions with policy-makers from the MOH and the insurance companies. Moreover, we communicated and wrote to these companies, as well as the Under-Secretary of treatment related to the MOH, and other private or public hospitals and academic institutes and explained to them the important role of CR and secondary prevention in return-to-work and improving patients’ quality of life. Furthermore, we sent them documentation from other countries where CR provision is national policy, and subsequently how patients are systematically referred to CR in these countries.

We persisted in our efforts until we reached an agreement in 2000. The MOH sent a circular to inform hospitals with cardiology or cardiovascular surgery departments that have CR programs that all components of CR will be covered by insurance companies. As a result CR attendance rates have gradually increased. The CRRC undertakes national studies on CR for monitoring and evaluation, to demonstrate the impact of this policy change for patient access, as well as patient health outcomes.

Qatar

CR services in Qatar are young and expanding. In 2000, two physiotherapists started providing services to patients with CVD who had been admitted to Hamad General Hospital in Doha. Soon the benefits of early physiotherapy in this patient group were appreciated, and budget for the recruitment of additional staff was approved. Nursing and occupational therapy also joined the “Cardiac Care Team” and education on CVD risk factors and home activities were included in the provided services. Educational materials were created and translated into several languages, including Hindi, Urdu, and Malayalam.

In 2009, and while the team included seven physiotherapists and three occupational therapists, a CR Planning Committee was formed in order to plan, develop, and implement a CR program in Hamad Medical Corporation, the principal public healthcare provider in Qatar. The committee’s work resulted to a proposal for the creation of a CR Program and coincided with the opening of the Heart Hospital, a hospital exclusively for patients with heart disease, in 2010. The proposal was approved by the founder of cardiology in Qatar and Director of Medical Education at that time, and a CR Department was founded in the Heart Hospital.

Staff recruitment followed and in 2011 the CR Department officially implemented phase 1 CR. The team expanded and new disciplines, such as dietetics, pharmacy, and speech therapy, were included. The phase 2 CR program was officially implemented in 2013, and provides CR services based on the Canadian,^[[1]](#footnote-1)^American^[[2]](#footnote-2)^, and European^[[3]](#footnote-3)^ guidelines for CR and secondary prevention.

One year later the working group of Qatar Association for Cardiovascular Prevention and Rehabilitation (QACPR) was created, as an official branch of the Gulf Heart Association-Qatar. Its primary goals include the development of the QACPR, the promotion of CR as an essential not optional service, the establishment of nationwide coverage of CR in Qatar, and the communication and collaboration with regional and international associations and healthcare providers.

Currently, the CR department is comprised of more than 30 staff members, and fosters an interdisciplinary approach to patient care. It is the sole provider of CR in the state of Qatar. In 2014, 85% of eligible patients were referred for CR; however, due to challenges in capacity and staffing, only 26% of the referred patients enrolled in a CR program. The department is continuously expanding in space and staff and it is expected to increase the absorption of eligible patients in the coming years. To this end, alternative program models, such as home-based and telephone-based CR programs are also being considered for implementation. At the same time, collaborations with external institutions, such as Qatar Diabetes Association, are being formed in order for CR phase 3 (community-based phase) to be initiated.

England

In England, advocacy for CR has resulted in the creation of a National Commissioning Guide and Tool-kit which is available at the following web link: <http://webarchive.nationalarchives.gov.uk/20130107105354/http://www.dh.gov.uk/en/Publicationsandstatistics/Publications/PublicationsPolicyAndGuidance/Browsable/DH_117504>

This package provides a tailored set of guidance, templates, tools and information to commission CR in a standardized fashion. The commissioning guide was mainly designed for Health Service commissioners in England, which are divided into 211 regional service commissioning groups, which serve a population of ~50 million people. The National Health Service (NHS) distributes money to these groups, which are then led by cooperatives of General Practitioners, who agree service contracts on behalf of their regional patient population.

The development of the CR commissioning guide was led by a dedicated service delivery support team (NHS Heart Improvement), from the Department of Health. The guide was written in collaboration with the national heart and cardiovascular health charity (The British Heart Foundation; <https://www.bhf.org.uk>) along with specialist representatives from the British Association for Cardiovascular Prevention and Rehabilitation ([www.bacpr.com](http://www.bacpr.com)) and other stakeholder groups, including the national heart patients association (The Cardiovascular Care Partnership UK; <http://www.bcs.com/pages/page_box_contents.asp?PageID=325>).

This first service commissioning guide for CR was produced in 2010, after years of evidence-based campaigning. It has been augmented by a more recent commissioning guide produced by England’s National Institute for Health Care Excellence (NICE CMG40, 2013; <http://www.nice.org.uk/guidance/cmg40>), along with complimentary guidance published in NICE’s myocardial infarction secondary prevention guide (CG172, 2013; <http://www.nice.org.uk/guidance/cg172>).

Success of the implementation of the commissioning pack has been evaluated both as a benefit to service commissioners and service providers (<http://www.cardiacrehabilitation.org.uk/docs/NACR_NHS_Improvement_audit.pdf> ).

With years of campaigning, these guides to commissioning as well as the release of national standards for delivering CR ([www.bacpr.com](http://www.bacpr.com)), in 2014 CR uptake reached almost 50% of eligible patients. This uptake of CR is 1.5 to 2.0 times greater than what is observed in other high-income countries (<http://www.cardiacrehabilitation.org.uk>).

United States

A sample of advocacy work by the American Association of Cardiovascular and Pulmonary Rehabilitation (AACVPR) includes the following:

1. Performance Measures for CR Referral: Beginning in 2005, AACVPR partnered with the American College of Cardiology and the American Heart Association to develop and test performance measures (i.e., quality metrics) for CR referral. These measures were aimed at improving CR referral and participation through the process of quality improvement and accountability. The CR referral performance measures were endorsed by the National Quality Forum, an organization that is a governmental-private partnership that identifies and endorses performance measures that may be potentially used by the Centers for Medicare and Medicaid Services (CMS) and other healthcare insurance carriers. The process of performance measurement development, testing, endorsement, and implementation is complex, long, and ever-changing. The impact of performance measures on CR utilization is still unclear, but one initial study suggests that they may have contributed to significant increases in CR referral in the United States from 2007, when the performance measures were officially endorsed and published in 2011 in the Journal of the American College of Cardiology (http://www.ncbi.nlm.nih.gov/pubmed/24768872)
2. Heart Failure as a covered indication for CR: AACVPR, in coordination with other partnering organizations, submitted scientific evidence supporting the benefit of CR participation for heart failure patients over a 10-year span. These efforts, along with a growing body of evidence in support of these efforts, led the U.S. government’s health insurance provider (CMS) to provide coverage for heart failure beginning in 2014.
3. Expansion of covered diagnoses for CR coverage: AACVPR submitted published, scientific evidence in 2001 to CMS, as they successfully petitioned CMS to expand coverage for CR to include additional diagnoses: heart valve repair/replacement, heart or heart/lung transplantation, and percutaneous coronary intervention. CMS granted this petition in 2006.
4. Demonstration projects with governmental or private insurance companies seeking clinical and/or cost-savings outcomes of CR: Government-sponsored demonstration projects are currently being carried out with CMS that are exploring the impact of bundled payment for the continuum of cardiovascular care for patients with heart failure and other forms of cardiovascular disease. CR services are an important part of the expected care received for the bundled payment.

In 2014, the government required that state health plans cover essential health benefits (EHB), defined as items and services in ten benefit categories. Two of those categories potentially related to CR are: (1) rehabilitative services and devices, and (2) preventive/wellness services and chronic disease management. The Maryland EHB Benchmark Plan is an example of a state that chose to include CR under required benefits.

1. Program Certification: Proliferation of CR programs nationally and internationally consequent to the development and promotion of voluntary program certification (quality improvement) and professional certification (individual practitioners) offered by the national professional organization AACVPR.

On a national level, hospitals competing for business have an incentive to promote a local certified CR program with certified professionals over others in the region that are not certified or cannot claim similar staff qualifications. An international example is South Korea which has embraced the opportunity to belong to the AACVPR, to model CR services in South Korea after those in the U.S., and to offer professional certification as this relatively new field emerges in that country.

1. Canadian Association of CR. 2009. Canadian Guidelines for CR and Cardiovascular Disease Prevention: Translating Knowledge into Action, 3rd edt. Available at www.cacr.ca/resources/guidelines.cfm [Accessed January 2015]. [↑](#footnote-ref-1)
2. American Association of Cardiovascular and Pulmonary Rehabilitation. 2013. Guidelines for CR and Secondary Prevention Programs, 5th edt. Champaign, IL: Human Kinetics. [↑](#footnote-ref-2)
3. Fifth Joint Task Force of the European Society of Cardiology; European Association of Echocardiography; European Association of Percutaneous Cardiovascular Interventions; European Heart Rhythm Association; Heart Failure Association; European Association for Cardiovascular Prevention & Rehabilitation; European Atherosclerosis Society; International Society of Behavioural Medicine; European Stroke Organisation; European Society of Hypertension; European Association for the Study of Diabetes; European Society of General Practice/Family Medicine; International Diabetes Federation Europe; European Heart Network. European Guidelines on cardiovascular disease prevention in clinical practice (version 2012): the Fifth Joint Task Force of the European Society of Cardiology and Other Societies on Cardiovascular Disease Prevention in Clinical Practice (constituted by representatives of nine societies and by invited experts). Eur J Prev Cardiol. 2012;19(4):585-667 [↑](#footnote-ref-3)
