# Supplementary material for: Advocacy for outpatient cardiac rehabilitation globally
Source: BMC Health Serv Res. 2016 Sep 6;16:471. doi: 10.1186/s12913-016-1658-1 (PMC5013580; doi:10.1186/s12913-016-1658-1)

Social Media
Magazines
Newspapers
Television
Posters
Video
Pamphlets
Blogs

Share the cause

Highlight the burden
Benefits of cardiac rehabilitation
Utilization of cardiac rehabilitation
Policy related to cardiac rehabilitation
Reimbursement for cardiac rehabilitation

Make speeches

Provide information
Educate on cardiac rehabilitation
Promote benefits of cardiac rehabilitation
Highlight underutilization of cardiac rehabilitation
Need for policy to support reimbursement

Contact decision makers

B AN ADVOCATE FOR CARDIAC REHABILITATION

Patient groups

Patient led awareness campaigns
Petition signing camps
Leading by example
Involve successful patients into cardiac rehabilitation program development

Implement programs

Promote cardiac rehabilitation in your hospital
Develop blanket referral systems to ensure all receive cardiac rehabilitation
Work with local professional organisations to include cardiac rehabilitation in their guidelines

Its your fight for Cardiac Rehabilitation!

International Council of Cardiovascular Prevention and Rehabilitation (ICCPR)

CONTACT

Email: globalcardiacrehab@gmail.com; Twitter: ICCPR_GlobalCR

Dr. Sherry Grace (sgrace@yorku.ca)-Chair

Dr. Aashish Contractor (ascontractor@gmail.com)-Vice-Chair

Dr. Gabriela Ghisi (gabriela.ghisi@gmail.com)-Secretary

International Council of Cardiovascular Prevention and Rehabilitation (ICCPR)

Advocacy for Cardiac Rehabilitation

A project of the International Council of Cardiovascular Prevention and Rehabilitation

To bring together national associations from around the world to harmonize efforts in promoting cardiovascular prevention and rehabilitation

To document consensus among national associations globally, regarding the internationally common core elements and benefits of cardiovascular disease prevention and rehabilitation.

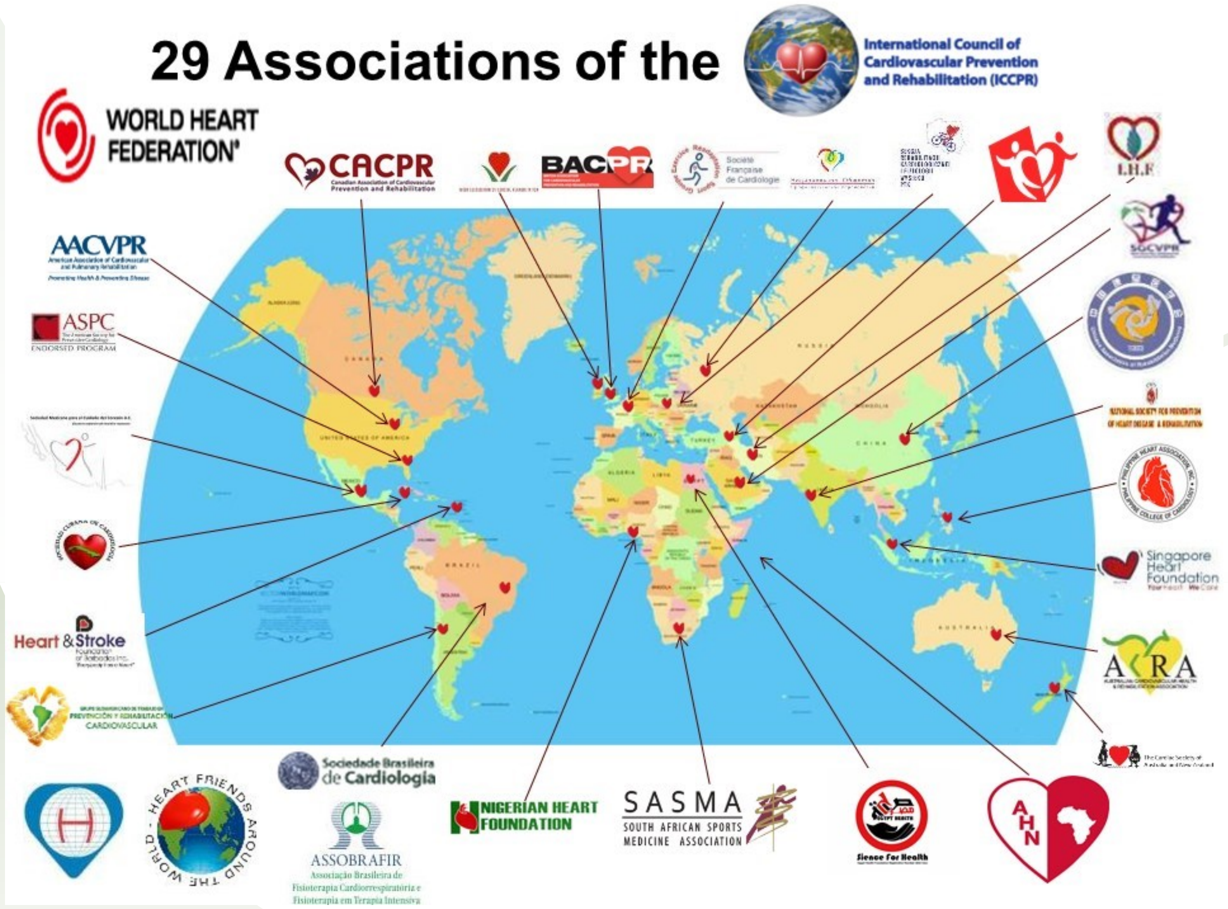

Abraham Samuel Babu, MPT, Francisco Lopez-Jimenez, MD, MSc, Wanrudee Isaranuwatchai, PhD, Artur Herdy, MD, Randal J. Thomas, MD; Jeffrey S. Hoch, PhD, Sherry L. Grace, PhD in conjunction with the International Council of Cardiovascular Prevention and Rehabilitation (ICCPR)

Why Cardiac Rehabilitation?

Improves outcomes

- Improved cardiovascular risk profile
- Better control of blood pressure, blood sugar and cholesterol
- Improves exercise capacity
- Facilitates faster return to work

Saves lives

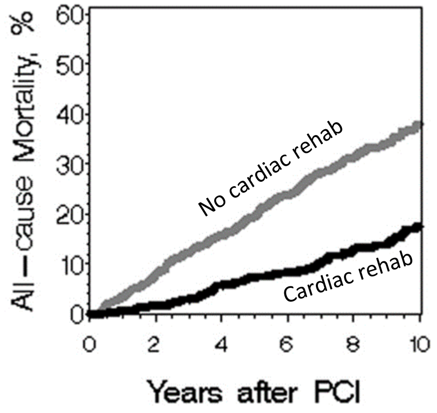

Economical

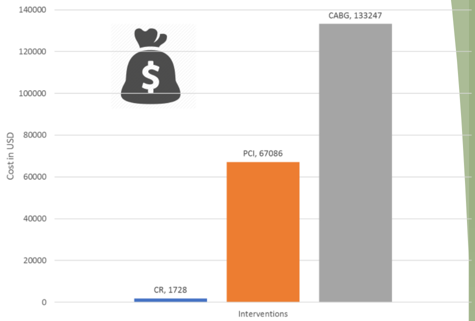

In reality…….

Only a few around the world receive it

Only ~40% are referred

55% of patients pay out-of-pocket

What is the solution??

Greater reimbursement

- From Government
- Greater coverage for CR sessions

Alternate models of delivery

- Home based rehabilitation

Greater awareness

- Improve awareness among patients, care-givers and health care professionals

Improved referral rates

- Facilitate automatic referrals to CR centers

Lobbying with Government for policy

Facilitate dialogues between stakeholders and policy makers to facilitate policy for promotion CR by enhancing reimbursement/ making it mandatory from National schemes

Social media and television

- Improve awareness by posting on social media
- Facilitate patient comments and experiences

How do we execute this?

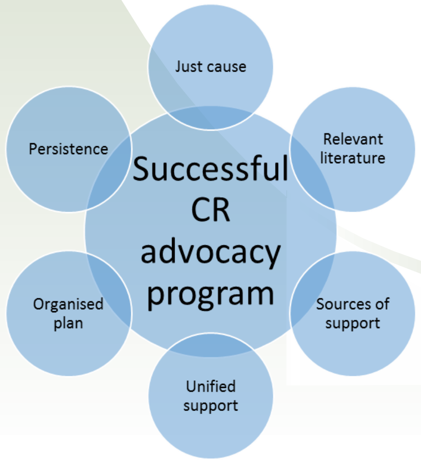

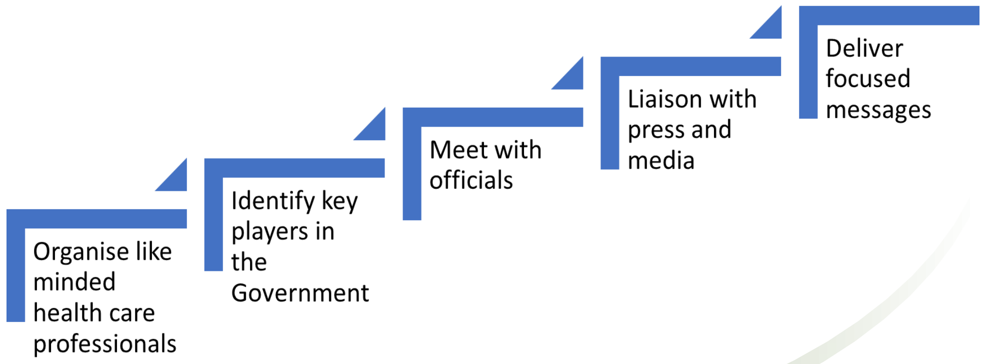

Supplement: Additional file 2: — Cardiac rehabilitation pamphlet. (PDF 1.09 mb) [file 12913_2016_1658_MOESM2_ESM.pdf]
