# Supplementary material for: Advocacy for outpatient cardiac rehabilitation globally
Source: BMC Health Serv Res. 2016 Sep 6;16:471. doi: 10.1186/s12913-016-1658-1 (PMC5013580; doi:10.1186/s12913-016-1658-1)
Supplement: Additional file 3: — Cardiac rehabilitation advocacy tool kit. (PDF 229 kb) [file 12913_2016_1658_MOESM3_ESM.pdf]

International Council of Cardiovascular Prevention and Rehabilitation (ICCPR)

CARDIAC REHABILITATION

ADVOCACY TOOL KIT

Prepared by:

Abraham Babu, PT (India), Francisco Lopez Jimenez (US / LAmerica) & Sherry L. Grace, PhD (Canada)

2016-2017

Note: The advocacy publication, report, this toolkit and pamphlet have been endorsed by ICCPR Council.

INTRODUCTION

The aim of this kit is to provide some more “hands-on” tools/advice to cardiac rehabilitation (CR) other organizations in advocating for CR reimbursement and delivery, as a complement to the ICCPR Advocacy Report and accompanying publication in *BMC Health Services Research* (Babu et al., 2016; see also the success stories in its' supplement online; https://bmchealthservres.biomedcentral.com/articles/10.1186/s12913-016-1658-1). The report provides the evidence basis to support CR advocacy, and outlines some approaches to go about advocating. The suggestions herein are based heavily on the WHF and Australian advocacy toolkits (see links at end of document).

EXECUTIVE SUMMARY OF HOW TO ADVOCATE FOR CR:

ICCPR recommends you consider the following 4 elements in your cardiac rehab advocacy campaign:

1. Organize a group of professionals with passion on CR that will plan the strategy to talk to the government.
2. Identify the key players in your government: the decision-makers and those who determine public policy for chronic diseases.
3. Meet with these elected officials and present the benefits of CR.
4. Work with social media and also the press to increase awareness; and share resulting releases with your government representatives and policy-makers.

HOW

Advocacy tools include social media, letters to potential payers, petitions / signature campaigns, and events. Ultimately you would like to have an in-person meeting with the individual who makes decisions on funding cardiac rehab.

WHO (more on this below)

It is imperative to learn who in your government makes decisions about funding healthcare services such as cardiac rehab (i.e., elected politicians, government bureaucrats in Ministry of Health?). Your goal will be to have an informed meeting with this decision-maker to discuss CR reimbursement.

Before approaching the decision-maker, consider who influences him or her. Do you have any contact with their primary care physician, and cardiologist of their family member, their religious leader, or influential business leader, philanthropic group or academic colleagues?

Engaging the public in your cause and ensuring they are informed can be helpful as their voices put pressure on decision-makers. This can include engaging the media and using social media. With regard to the former, you should search for which reporters have previously done stories on heart disease. You can pitch a media release to these journalists. Or you can write an Op-Ed to leading newspapers. You can also engage the medical community to writing a Letter to the Editor in an influential journal, and writing a piece in their newsletters or posting to their website.

Consider who will deliver the advocacy message to the decision-maker. Likely the head of major CR, cardiac or medical associations, patient groups, and respected academics would be most persuasive. The more influential groups such as these that you have communicating the same message, the stronger your impact will be.

WHEN

Advocacy is needed now, but consider taking advantage of opportunities when the message of CR may be better received. This can include the 2018 High Level Meeting on non-communicable diseases (which includes cardiovascular disease). National governments are committed to working towards Sustainable Development Goal 3 on health, and cardiac rehab can help them achieve 25x25 (http://www.world-heart-federation.org/no_cache/what-we-do/global-advocacy/25-by-25/) or 1/3 reduction in premature mortality from cardiovascular diseases by 2030 (see: http://www.world-heart-federation.org/what-we-do/global-advocacy/agenda-2030/).

This could also include World Heart Day (Sept 29), national cardiovascular conferences, or before key government meetings.

WHAT (more on this below)

While decision-makers may be aware about the toll of cardiovascular disease, they are likely not familiar with cardiac rehab as an established, cost-effective and impactful model of care which optimizes the health and well-being of the many heart patients. They may know someone with heart disease, and this can help to personalize the cause. Indeed, using patient case studies or narratives can really emphasize the evidence about the need for and benefits of cardiac rehab.

To prepare, do some research to understand if there has been any previous CR advocacy, and by whom. Also be sure to know what the government's policies regarding cardiac rehab, are and what their policy goals are. You want to frame the cardiac rehab message as a means to meet their goals.

Given economic realities, be sure to focus on the economic case for cardiac rehab as outlined in the accompanying report. Do your best to have information about the cost to deliver cardiac rehab in your country (see costing template in appendix at end of this document to get you started), and how many patients need our services. This should include information on how much cardiac rehab is currently available and the proportion of patients you are reaching (and any local data you have on the beneficial impact of cardiac rehab on these patients). You can focus on how private insurance could be brought in to support financing of cardiac rehab, and how this is successfully done in other countries (see our success stories).

Leave some materials with the decision-maker so they can understand more about the need for cardiac rehab (i.e., facts, infographics; e.g., https://www.heartfoundation.org.au/images/uploads/publications/HF.Cardiac_Rehab_Factsheet_WEB HR.pdf).

Key Messages:

Heart disease is one of the most common conditions worldwide, impacting people in their most productive years. Patients with heart disease are at great risk of death and disability, and therefore need a comprehensive approach to manage this. Cardiac rehab is a proven approach to ensure the health and well-being of patients, by delivering structured exercise training and risk factor management.

By ensuring cardiac rehab is available to heart patients and is covered, the benefits to you are: national reductions in cardiac morbidity and mortality; reduced healthcare utilization and associated costs; system of care to manage cardiac disease.

The cost of delivering cardiac rehab is significantly smaller than the cost of inaction. We must act now to ensure a heart healthy future for all.

To recap: **Key Points to Convey:**

- Introduce the burden of cardiovascular disease on health, family and the economy
- Provide a case scenario/ story of an average, middle-class family member suffering from an acute coronary event
- Emphasize the cost of treatment using supportive data (Provide either full paper or link to full paper)
- Describe our cost-saving intervention – cardiac rehabilitation
- Describe the benefits of cardiac rehab: reduced death, disability and improved quality of life. Highlight the importance of return-to-work and how cardiac rehabilitation can facilitate this
- Provide information on how cardiac rehabilitation is so poorly available and used, using supportive data (Provide either full paper or link to full paper)
- Talk about our cause to ensure cardiac rehab is available at no cost to patients

Here are some suggestions and considerations for engaging with these various stakeholders:

1. **Engaging the public, healthcare professionals and insurance companies:**
   The ICCPR is making use of social networking to promote cardiac rehabilitation delivery and reimbursement. Take the time to join us on Twitter (@ICCPR_GlobalCR) and contribute to this campaign! Social media is free and has broad reach.

   Posters and pamphlets (see attached and links at end of document) promoting the need for cardiac rehabilitation can be posted at professional conferences and gatherings to increase awareness. These can also be circulated to insurance companies.

   Here are some other resources to share: Patient information on cardiac rehab…

- from the American Heart Association: http://www.heart.org/HEARTORG/Conditions/More/CardiacRehab/Cardiac-Rehab_UCM_002079_SubHomePage.jsp
- And from the Heart Foundation of Australia: https://www.heartfoundation.org.au/your-heart/living-with-heart-disease/what-is-cardiac-rehabilitation

2. **Engaging patient groups**
   Patients are the strongest advocates for cardiac rehabilitation. Where patient groups exist, it will be beneficial to engage them to join your advocacy efforts.

   How can they do this?
   - Organizing walks for cardiac patients who have undergone cardiac rehabilitation
   - Submitting signed petitions from patients and supporters (caregivers or health care professionals) to government

3. **Engaging government, policy-makers and health care administrators**
   Get in touch with members of government and educate them on the need for cardiac rehabilitation. A sample letter you can work from is found at the end of this toolkit.

   Important aspects to be kept in mind while contacting and communicating with members of government are:
   - Their time is very limited
   - Personalise the message – AVOID using the same letter or speech to all of them
     - What does government stand to gain by supporting cardiac rehab?
     - How will government better serve the people?
   - Describe a clear benefit that would result from their support
   - Try to secure a commitment to further explore cardiac rehab reimbursement with your organization

   Most importantly……. STAY ON POINT! (we want policy for CR reimbursement and standard delivery for all)

CONCLUDING REMARKS

It is important to note that no advocacy approach is universally applicable. Use any levers, and consider barriers and enablers. We hope elements of this toolkit can be selected and adapted to suit your context.

If you have further suggestions or feedback, please be in touch with us at globalcardiacrehab@gmail.com. We wish you success in your efforts to secure cardiac rehab reimbursement and broader access for all. ICCPR will continue to advocate at the international level.

SAMPLE LETTER FOR MINISTRIES OF HEALTH / GOVERNMENT OFFICIALS

{logo}

{Date}

Dear Minister………………………………..,

I am writing on behalf of the International Council of Cardiovascular Prevention and Rehabilitation (ICCPR) and [……..] to ask for your support in the delivery of cardiac rehabilitation. At your earliest convenience, we request a meeting with your office to discuss cardiac care, and how we the heart health community can be of assistance.

Cardiovascular disease is the leading cause of death in the world, and is responsible for nearly half of the non-communicable disease burden. In (country name), the burden is……………………………. (some information may be available here: http://www.who.int/nmh/media/ncd-progress-monitor/en/) Heart disease burdens individuals in their most productive years; this disability and illness at early ages is largely preventable.

The World Economic Forum cites non-communicable diseases such as heart disease as one of the top risks to the global economy. Globally, heart diseases cost $473.9 billion USD in 2010. The total cost of heart diseases, including lost productivity, is expected to rise to $1,044 billion USD in 2030 –an increase of 22%. (Add #s re impact on national economy if available)

This is why there is a pressing need for cost-effective methods to mitigate this burden such as cardiac rehabilitation. It is a team-based approach to prevent and control risk factors for cardiovascular disease. Patients who participate in cardiac rehab have 26% lower death rates, are more likely to return-to-work and are less likely to use healthcare services.

Unfortunately we only have xx cardiac rehab programs in {country}, and only xx% of heart patients access these services. The ICCPR, along with (our local organization) are seeking support from the Government **to increase delivery of cardiac rehab, and to ensure reimbursement of cardiac rehab services**.

We know you want to provide accessible, affordable, quality cardiovascular care, comprised of the recommended prevention and treatment services. Cardiac rehab is a key element of this care. By supporting cardiac rehab, our country will be better able to meet our commitments toward the Sustainable Development Goals /2030 Agenda (particularly # 3 Health, target 4 to reduce by one-third premature mortality from non-communicable diseases through prevention and treatment). We must devote appropriate resources to curb the burden of heart disease.

Supporting documents are provided to help you better understand our cause. Thank you for your consideration, and we look forward to discussing this pressing issue with you soon.

Best wishes,

{Insert Name}

{Insert title}

{Insert contact information}

APPENDIX: CR costing template

This costing template is provided to facilitate estimation of the cost to run a CR program. The elements listed are based on the minimum requirements for CR as forwarded in the ICCPR Consensus Statement on CR Delivery in Low-Resource Settings, and can be modified to fit with your regional realities.

To create a more detailed CR business case, see the resources here: http://webarchive.nationalarchives.gov.uk/20130107105354/http://www.dh.gov.uk/en/Publicationsandstatistics/Publications/PublicationsPolicyAndGuidance/Browsable/DH_117507, and costing tool template with guidance here: http://webarchive.nationalarchives.gov.uk/20130107105354/http://www.dh.gov.uk/en/Publicationsandstatistics/Publications/PublicationsPolicyAndGuidance/Browsable/DH_117657

Element	Quantity	Unit cost	Cost
**Personnel**<br>Community healthcare worker<br>Staff 1<br>Staff 2<br>Others			
**Materials**<br>Blood Pressure measuring device or stethoscope<br>Measuring tape (loose)<br>Measuring tape (wall-mounted)<br>Weigh scale<br>Urine test strips<br>Home-made weights for resistance training<br>Paper and pencils<br>Secure and confidential filing<br>Other			
**Other**<br>Facility/space<br>Overhead<br>Other			

USEFUL LINKS & RESOURCES

Heart Foundation of Australia's CR Advocacy Toolkit: https://www.heartfoundation.org.au/images/uploads/publications/Heart_Foundation_A4_Advocacy_ToolKit_Brochure_WEBHR.pdf

Heart Foundation of Australia's CR Advocacy Pamphlet: https://heartfoundation.org.au/images/uploads/publications/Improving-the-delivery-of-cardiac-rehabilitation.pdf

Heart Foundation of Australia's CR Advocacy Plan: http://www.heartlungcirc.org/article/S1443-9506(15)00075-X/abstract

ACRA CR Advocacy Presentation Slides: http://www.acra.net.au/wp-content/uploads/2015/08/Cardiac-rehabilitation-the-Heart-Foundations-strategy-to-drive-change-through-advocacy-Rachel-Neumann.pdf

WHF Advocacy Resources, including 3 toolkits, factsheets and slides: http://www.world-heart-federation.org/what-we-do/global-advocacy/advocacy-resources/

AACVPR Advocacy Resources: https://www.aacvpr.org/Advocacy

- See also their strategies to increase CR use for healthcare providers (scroll down to Turnkey Enrollment/Adherence Strategy Documents): http://www.aacvpr.org/R2R

The AACVPR holds an annual day of advocacy directed to legislators in Washington. More information can be found at: https://www.aacvpr.org/Advocacy/Advocacy-Day-on-the-Hill.

ICCPR Advocacy Resources: http://globalcardiacrehab.com/advocacy/
